# A holistic intervention program for children from low socioeconomic status families

**DOI:** 10.3389/fpsyg.2014.00797

**Published:** 2014-07-25

**Authors:** Jonathan S. E. Tan, Hwajin Yang, Sujin Yang

**Affiliations:** School of Social Sciences, Singapore Management UniversitySingapore

**Keywords:** cognitive development in children of low-socioeconomic families, holistic intervention program, family context, home environment, early intervention program

There is considerable evidence that children from families with low socioeconomic status (SES) are at risk of profound delays in cognitive development and educational achievement. Scholars and policy makers have therefore sought to identify the potential causes of these problems and to design interventions to narrow this achievement gap. With this goal, Neville et al. (2013) developed a family-based intervention (PCMC-A) to improve neurocognitive functions supporting selective attention in low-SES preschoolers. Involving both parents and children in the training, they demonstrated that the PCMC-A significantly improved nonverbal IQ, receptive language, neurocognitive functions supporting early attentional processing, parent-reported social skills, and parent-child interactions, and reduced parenting stress. Given that previous studies have focused on the training of children, it is noteworthy that Neville et al. examined factors related to not only children but also parents (e.g., parents' stress regulation and contingency-based discipline) and the home environment (e.g., parent-child interaction, parents' language use with the child, and facilitation of child attention).

Although the authors' findings are interesting and their contributions are remarkable, the family-related factors chosen for the study are far from satisfactory, as the study did not adequately consider the broader family context, which is crucial in capturing the richer dimensions of SES. Our primary goal is therefore to draw attention to the challenges posed by the family-based intervention of Neville et al. In particular, we recommend that future research consider risk factors such as personal resilience, as well as maternal and environmental factors, as they have been shown to affect cognitive development in children from low-SES families (for a review, see Bradley and Corwyn, 2002; Evans, 2004, 2006).

Specifically, resilience—a dynamic process wherein individuals display positive adaptation despite significant adversity or trauma (Luthar and Cicchetti, 2000)—is a crucial index of individual differences in the fundamental adaptive system. Often overlooked, it has nonetheless been proven to be a positive moderator for many low-SES children who do well despite the odds (Knitzer and Perry, 2009). Early research on resilience—assessed by children's attachment security or social competence (Luthar and Cicchetti, 2000; Masten, 2001)—has largely emphasized the importance of personality traits and active coping strategies that help children overcome adversity and the risk factors associated with low SES (Bradley and Corwyn, 2002). Thus, future interventions should promote resilience by focusing on self-regulation skills that are essential in facilitating children's adaptive abilities, such as self-control, social competencies, and emotion regulation. In fact, the effectiveness of self-regulation has been demonstrated in various intervention programs (Greenberg, 2006; Diamond et al., 2007; Bierman et al., 2008; Raver et al., 2011).

Moreover, future family interventions must include maternal risk factors. The literature has consistently shown that maternal depression and substance abuse (e.g., cocaine, tobacco, or alcohol) have been linked with severe consequences for cognitive development (Petterson and Albers, 2001; Shankaran et al., 2007). Furthermore, because low-SES infants are generally more prone to experience these maternal risk factors than their high-SES counterparts, the consequences are usually more pronounced in children from low-SES families (Parker et al., 1988; McLoyd, 1998). Hence, it is important to screen mothers with either depressive symptoms or previous history of substance abuse. Alternatively, future family interventions should consider including a self-care program (e.g., relaxation, social skills, personal development and recreational activities, or marital adjustment), caregiving practices (e.g., co-parenting or child-care resources), or substance abuse treatment (e.g., counseling or relapse prevention), all of which are known to improve maternal mental well-being.

Finally, there is no doubt that chronic noise exposure, crowded housing (calculated by the number of people per room), substandard housing (low-quality construction or lack of privacy, cleanliness, tidiness, or children's resources), and poor neighborhood quality are prevalent among low-SES homes (for a review, see Evans, 2004, 2006). An increasing body of evidence suggests that these factors are an important cause of decreased cognitive functioning in children, given that they disrupt activities such as studying, exploration, and play. For instance, children exposed to transportation noise (principally aircraft) have been found to manifest significant delays in the development of reading ability when SES is controlled for (Evans and Hygge, 2007). Residential crowding has been shown to negatively affect not only child-parent interactions (Bartlett, 1998) but also verbal, perceptual, and quantitative performance during early childhood (Gottfried and Gottfried, 1984). Lastly, children from homes with irregular schedules for homework, bedtime, etc., have shown deficits in cognitive development (Petrill et al., 2004). Given the above, it is noteworthy that previous interventions such as income intervention programs and residential mobility programs—which provide low-income families with tenant-based rental subsidies—have been successful in improving schooling outcomes and reducing problem behaviors in low-SES children (Gennetian and Miller, 2002; Johnson et al., 2002). Taken together, family-based interventions require a holistic and multilevel approach that examines the extent to which a broad developmental context (i.e., individual differences combined with home and parental factors) modulates intervention outcomes for low-SES children, as these factors can increase the efficacy of programs fostering cognitive development in low-SES children.

In conclusion, given the detrimental impact of SES—a multifaceted construct—on cognitive development during early childhood, an integrative approach to intervention programs for low-SES children is warranted. In addition, understanding the specific mediators involved would improve future intervention programs by allowing greater control and precision. The concept of precision is especially critical, given the difficulty of separating the effects of low SES and multiple co-occurring variables on child development (Bradley and Corwyn, 2002). The effectiveness of early intervention programs can be further supported by the formulation of specific national and state policies tailored to address the high-risk factors that plague low-SES families, such as complications during birth, maternal mental health, and housing and neighborhood conditions. Economists have also demonstrated that increased societal investment in early intervention programs improves cognitive ability among low-SES children, which increases overall societal welfare in the long run (Heckman, 2006). Clearly then, the development and refinement of intervention programs, which can yield substantial benefits for both low-SES children and society as a whole, is essential.

## Conflict of interest statement

The authors declare that the research was conducted in the absence of any commercial or financial relationships that could be construed as a potential conflict of interest.
